# The impact of geriatric characteristics and comorbidities on distant metastases and other cause mortality in older women with non-metastatic breast cancer treated with primary endocrine therapy

**DOI:** 10.1007/s10549-023-07029-4

**Published:** 2023-07-21

**Authors:** M. E.C. Waaijer, A. A. Lemij, A. Z. de Boer, E. Bastiaannet, F. van den Bos, M. G.M. Derks, J. R. Kroep, G. J. Liefers, J. E.A. Portielje, N. A. de Glas

**Affiliations:** 1 Department of Gerontology and Geriatrics, Leiden University Medical Center, Leiden, the Netherlands; 2 Department of Surgery, Leiden University Medical Center, Leiden, the Netherlands; 3 Department of Medical Oncology, Leiden University Medical Center, Post zone C7-Q, P.O. Box 9600 RC, Leiden, the Netherlands

**Keywords:** Breast cancer, Geriatric oncology, Primary endocrine therapy

## Abstract

**Introduction:**

In recent years, primary surgical treatment of older women with non-metastatic breast cancer has decreased in favor of primary endocrine therapy (PET). PET can be considered in women with a remaining life expectancy of less than five years. The aim of this study was to (1) assess the risk of distant metastases and other cause mortality over ten years in women aged 65 and older with stage I-III breast cancer treated with PET, (2) whether this was associated with geriatric characteristics and comorbidities and to (3) describe the reasons on which the choice for PET was made.

**Methods:**

Women were included from the retrospective FOCUS cohort, which comprises all incident women diagnosed with breast cancer aged 65 or older between January 1997 and December 2004 in the Comprehensive Cancer Center Region West in the Netherlands. We selected women (N = 257) with stage I-III breast cancer and treated with PET from this cohort. Patient characteristics (including comorbidity, polypharmacy, walking, cognitive and sensory impairment), treatment and tumor characteristics were retrospectively extracted from charts. Outcomes were distant metastasis and other cause mortality. Cumulative incidences were calculated using the Cumulative Incidence for Competing Risks method (CICR); and subdistribution hazard ratios (SHR) were tested between groups based on age, geriatric characteristics and comorbidity with the Fine and Gray model.

**Results:**

Women treated with PET were on average 84 years old and 41% had one or more geriatric characteristics. Other cause mortality exceeded the cumulative incidence of distant metastasis over ten years (83 versus 5.6%). The risk of dying from another cause further increased in women with geriatric characteristics (SHR 2.06, p < 0.001) or two or more comorbidities (SHR 1.72, p < 0.001). Often the reason for omitting surgery was not recorded (52.9%), but if recorded surgery was omitted mainly at the patient’s request (18.7%).

**Discussion:**

This study shows that the cumulative incidence of distant metastasis is much lower than other cause mortality in older women with breast cancer treated with PET, especially in the presence of geriatric characteristics or comorbidities. This confirms the importance of assessment of geriatric characteristics to aid counseling of older women.

**Supplementary Information:**

The online version contains supplementary material available at 10.1007/s10549-023-07029-4.

## Background

Around thirty per cent of women diagnosed with breast cancer is older than seventy [1]. In general, older individuals are highly variable with respect to comorbidities, functional and cognitive capabilities and social support system. Also, older individuals with cancer tend to value quality of life over longevity [2]. These factors can influence treatment decisions in older women with breast cancer. In the last decade, surgery has been more frequently omitted in older women with operable breast cancer and treatment with primary endocrine therapy (PET) has increased [3–5].

There are several reasons for choosing PET over primary surgery [3]. Women with more comorbidities have a higher rate of non-breast cancer related causes [6–8] and may not live long enough to develop distant metastasis. This holds especially true for those with low-risk breast cancer and a high other cause mortality risk, for example women with stage I breast cancer and severe dementia. This risk of dying from another cause than breast cancer (other cause mortality) can influence both patient and doctor preferences in breast cancer treatment. Current recommendations for treating older women with breast cancer (SIOG/EUSOMA) state that PET should only be offered to older women with estrogen receptor (ER) positive tumors who have an estimated life expectancy of less than five years, and are unfit for or refuse surgery [9]. Prediction tools are available to predict risk of recurrence and other cause mortality in older women with cancer to aid informing and advising older women in this treatment decisions [10–12].

In this study we aim to assess the ten year risk of distant metastases and other cause mortality in women aged 65 and older with stages I-III breast cancer treated with PET, and whether this was associated with geriatric characteristics and comorbidities. We further aim to describe treatment preferences.

## Methods

### Study population

Data was used from the retrospective FOCUS cohort study (Female breast cancer in the elderly; Optimizing Clinical guidelines Using clinic-pathological & molecular data), which comprises all incident women diagnosed with breast cancer aged 65 or older between January 1997 and December 2004 in the Comprehensive Cancer Center Region West in the Netherlands (N = 3672). This cohort, which was previously described in detail [13], was derived of the National Cancer Registry in the Netherlands containing all newly diagnosed tumors. The study was approved by the scientific board of the Netherlands Cancer Registry. For this study, we included women who had stage I-III breast cancer. We defined PET as not receiving primary surgery but being treated with endocrine therapy (tamoxifen or an aromatase inhibitor). In this time period, patients were not treated with neoadjuvant endocrine therapy, so we believe that this definition covers all patients who were treated with PET.

### Patient characteristics and treatments

Charts were reviewed by trained personnel and data was retrospectively extracted on tumor characteristics (histology, TNM stage), treatments, comorbidity (using ICD10 coding) and geriatric characteristics (walking impairment – defined as walking difficulties or use of walking aid noted in the chart, cognitive impairment – defined as history of dementia or cognitive impairment noted in the chart, sensory impairment - defined as positive if using a hearing aid or if poor vision was noted in the chart, and polypharmacy – defined 5 or more medications in use). If data were missing, they were classified as “missing” category within the variable. Age categories were defined as: 65 to 79 years (N = 81), and 80 years and older (N = 177). Geriatric characteristics were classified as having no geriatric characteristic present or 1 or more. Comorbidities were grouped in 0–1 and 2 or more comorbidities. Two additional categories combined the age groups with geriatric characteristic or comorbidities categories.

### Outcomes

Longitudinal outcomes were occurrence of distant metastasis and mortality from other causes than breast cancer defined as death without a distant metastasis. Follow-up data on mortality was acquired through linking the data from the National Cancer Registry with municipal population registries, until January 1st 2013. Data on the outcome occurrence of distant metastasis was extracted by trained personnel from medical charts. We give the outcome data over a time period of ten years, because a substantial number of women in this cohort survived past the recommended five years life expectancy to consider PET. Reasons for omitting surgery were recorded in categories (mental condition, physical condition, both mental and physical condition, patient preference, age, inoperable, other diagnosis determines prognosis) or in free text for other reasons. In case e.g. “mental condition” was mentioned together with “wish patient” the first reason was used.

### Statistics

The cumulative incidences of distant metastasis and other cause mortality (death in absence of distant metastasis) were calculated using the Cumulative Incidence for Competing Risks method (CICR) [14, 15]. We calculated cumulative incidences for the whole cohort, and separate cumulative incidences for different groups based on age categories, geriatric characteristics and comorbidities. The cumulative incidences between groups based on age, geriatric characteristics and comorbidities were tested using the Fine and Gray model. Analyses were adjusted for TNM stage and age. Analyses were performed in IBM SPSS Statistics version 25 and STATA version 14.0.

## Results

Table 1 shows the characteristics of the women in our study. Women treated with PET were on average 84 years old (to compare, the average age of women in this cohort that did choose surgery was 75.7 years). Polypharmacy was present in 14.4%. 14.8% of the women had cognitive impairment, 14.8% had a walking impairment, 18.7% a sensory impairment. Cumulating these geriatric characteristics results in 41.2% of the women treated with PET that have at least one geriatric characteristic present. Furthermore two or more comorbidities were present in 42.0% of the women. The TNM stages were distributed as follows: 0–1.2%, I − 19.8%, II- 34.2%, III – 16.3% and also 28.4% missing a TNM classification in their chart.

**Table 1:** Characteristics of women with non-metastatic breast cancer treated with PET

	Number (%) / Median (IQR)
	N = 257
**Patient related**	
Age	84.2 (77.7; 89.1)
Age categories	
65–69	18 (7.0)
70–74	22 (8.6)
75–79	41 (16.0)
80–84	57 (22.2)
85–89	71 (27.6)
90+	48 (18.6)
Geriatric characteristics	
Walking impairment	38 (14.8)
Polypharmacy	37 (14.4)
Cognitive impairment	38 (14.8)
Sensory impairment	48 (18.7)
1 or more geriatric characteristics	106 (41.2)
Comorbidities	
0–1 comorbidities	149 (58.0)
2 or more comorbidities	108 (42.0)
**Tumor related**	
TNM stage	
0	3 (1.2)
I	51 (19.8)
II	88 (34.2)
III	42 (16.3)
Missing	73 (28.4)
Grade	
1	5 (1.9)
2	19 (3.9)
3	5 (1.9)
Missing	237 (92.2)
Morphology	
Ductal	122 (47.5)
Lobular	23 (8.9)
Other/missing	112 (43.6)
ER/PR	
Positive ER or PR	86 (33.5)
Negative ER and PR	12 (4.7)
Missing	159 (61.9)

Frequencies in no. (%), no. (%), or median (interquartile range, IQR). N/a = not applicable

**Fig. 1:**
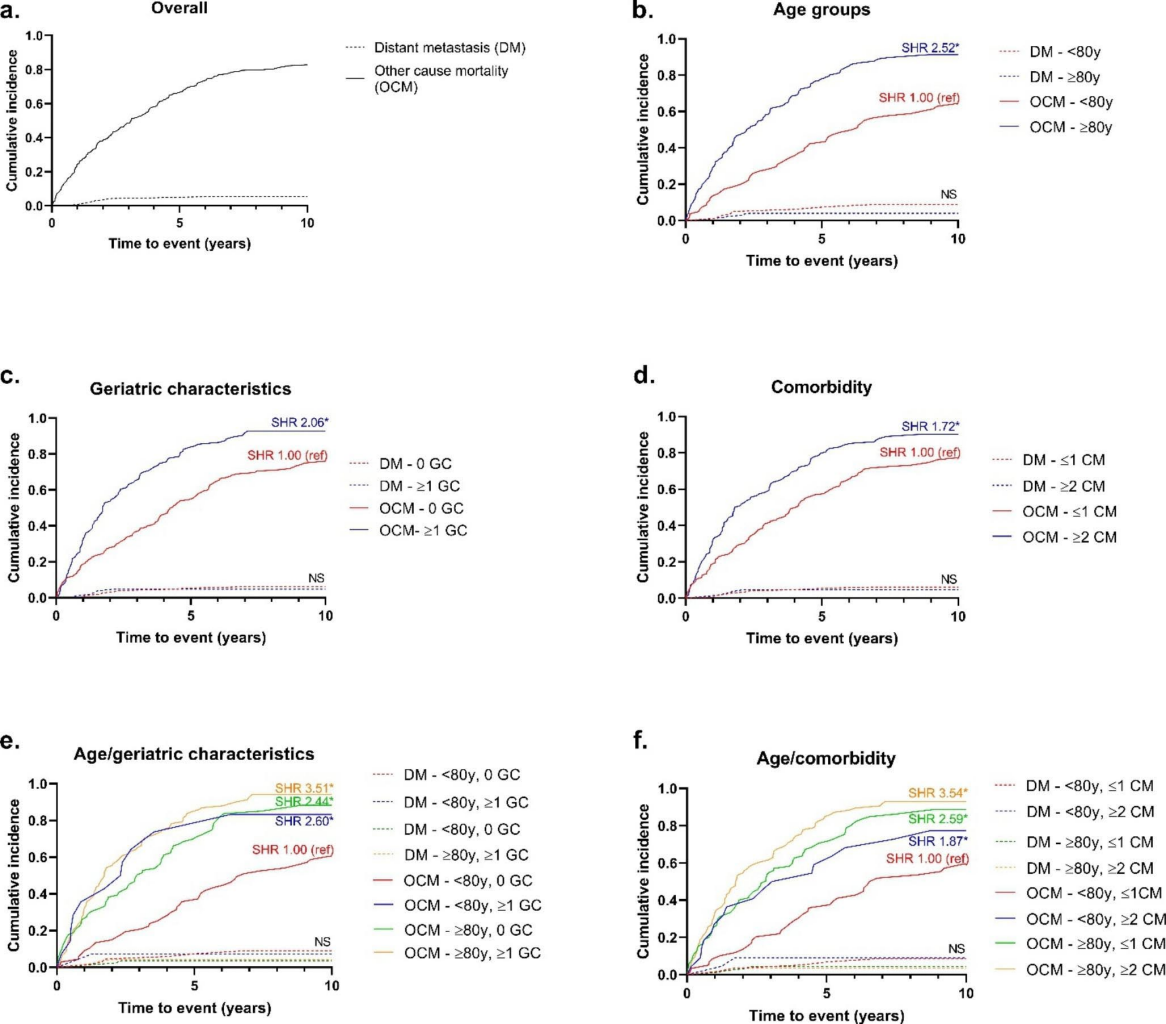
Ten years risk of distant metastasis and other cause mortality in women with non-metastatic breast cancer treated with PET, dependent on age, geriatric characteristics and comorbidity SHR: subdistribution hazard ratio. Ref: reference. DM: distant metastasis. OCM: other cause mortality. GC: geriatric characteristic, y: years. *: P ≤ 0.05, NS: not significant. Fine and Gray analyses were used for calculation of SHRs, results from the crude model are shown

Figure 1 shows the cumulative incidences (over ten years) of distant metastasis and of other cause mortality (death without distant metastasis), comparing different age groups, geriatric characteristics and corresponding subdistribution hazards (SHR’s) are presented in Table 2. Overall, the cumulative incidence of distant metastasis was 5.55% (95%CI 3.16–8.80%) whereas the incidence of other cause mortality was 82.7% (95%CI 77.3–87.0%) (Fig. 1a). Cumulative incidences of distant metastasis were not associated with age, number of geriatric characteristics or comorbidities.

**Table 2:** Subdistribution hazard ratios (SHR) of distant metastasis and other cause mortality in women with non-metastatic breast cancer treated with PET, dependent on age, geriatric characteristics and comorbidity

	Distant metastatasis - SHR (95% CI)			Competing event - SHR (95% CI)		
	Model 1	Model 2	Model 3	Model 1	Model 2	Model 3
Age						
65–79 years	1.00 (ref)	1.00 (ref)	na	1.00 (ref)	1.00 (ref)	n/a
≥80 years	0.46 (0.16–1.31)	0.39 (0.14–1.12)	n/a	2.52 (1.83–3.46)	2.39 (1.70–3.34)	n/a
Geriatric characteristics						
No walking impairment	1.00 (ref)	1.00 (ref)	1.00 (ref)	1.00 (ref)	1.00 (ref)	1.00 (ref)
Walking impairment	0.95 (0.21–4.24)	0.93 (0.20–4.27)	1.10 (0.22–5.56)	1.74 (1.22–2.50)	1.72 (1.17–2.53)	1.58 (1.07–2.33)
No cognitive impairment	1.00 (ref)	1.00 (ref)	1.00 (ref)	1.00 (ref)	1.00 (ref)	1.00 (ref)
Cognitive impairment	0.43 (0.06–3.28)	0.45 (0.06–3.59)	0.57 (0.06–5.05)	2.15 (1.47–3.15)	2.09 (1.42–3.06)	1.87 (1.30–2.68)
<5 medications per dag	1.00 (ref)	1.00 (ref)	1.00 (ref)	1.00 (ref)	1.00 (ref)	1.00 (ref)
≥5 medications per day	1.67 (0.46–6.05)	1.76 (0.47–6.64)	2.09 (0.55–7.94)	1.52 (1.05–2.22)	1.43 (0.96–2.11)	1.24 (0.81–1.90)
No sensory impairment	1.00 (ref)	1.00 (ref)	1.00 (ref)	1.00 (ref)	1.00 (ref)	1.00 (ref)
Sensory impairment	0.73 (0.16–3.31)	0.78 (0.17–3.63)	0.93 (0.75)	1.60 (1.15–2.24)	1.51 (1.07–2.13)	1.34 (0.95–1.90)
Total geriatric characteristics 0	1.00 (ref)	1.00 (ref)	1.00 (ref)	1.00 (ref)	1.00 (ref)	1.00 (ref)
Total geriatric characteristics ≥ 1	0.79 (0.26–2.37)	0.81 (0.25–2.61)	1.05 (0.31–3.54)	2.06 (1.55–2.74)	1.97 (1.46–2.63)	1.66 (1.23–2.25)
Comorbidities						
0–1 comorbidities	1.00 (ref)	1.00 (ref)	1.00 (ref)	1.00 (ref)	1.00 (ref)	1.00 (ref)
≥2 comorbidities	0.76 (0.25–2.27)	0.74 (0.24–2.28)	0.88 (0.24–3.21)	1.72 (1.31–2.28)	1.60 (1.20–2.12)	1.47 (1.11–1.96)
Age/geriatric characteristics						
65–79 years and 0 geriatric characteristics	1.00 (ref)	1.00 (ref)	n/a	1.00 (ref)	1.00 (ref)	n/a
65–79 years and ≥ 1 geriatric characteristics	0.83 (0.10–7.03)	0.91 (0.19–8.48)	n/a	2.60 (1.21–5.60)	2.46 (1.16–5.22)	n/a
>80 years and 0 geriatric characteristics	0.40 (0.10–1.60)	0.34 (0.08–1.37)	n/a	2.44 (1.68–3.56)	2.32 (1.56–3.45)	n/a
>80 years and ≥ 1 geriatric characteristics	0.49 (0.14–1.72)	0.43 (0.11–1.65)	n/a	3.51 (2.41–5.12)	3.35 (2.24–5.01)	n/a
Age/comorbidities						
65–79 years and 0–1 comorbidities	1.00 (ref)	1.00 (ref)	n/a	1.00 (ref)	1.00 (ref)	n/a
65–79 years and ≥ 2 comorbidities	1.10 (0.21–5.67)	0.98 (0.16–5.99)	n/a	1.87 (1.03–3.39)	1.76 (0.95–3.26)	n/a
>80 years and 0–1 comorbidities	0.53 (0.14–1.97)	0.44 (0.12–1.60)	n/a	2.59 (1.75–3.83)	2.49 (1.63–3.80)	n/a
>80 years and ≥ 2 comorbidities	0.41 (0.10–1.71)	0.34 (0.08–1.52)	n/a	3.54 (2.37–5.29)	3.11 (2.14–5.12)	n/a

SHR: subdistribution hazard ratio. 95% CI: 95% confidence interval. Ref: reference. N/a: not applicable. Fine and Gray analyses: model 1 - crude model; model 2 - as model 1 plus TNM stage; model 3 - as model 2 plus age

The cumulative incidence of other cause mortality was 91.2% (95%CI 85.7–94.7%) for women aged 80 and older, versus 64.3 (95%CI 52.6–73.9%) for women aged 65–79 years (Fig. 1b. Model 1: SHR 2.52, 95%CI 1.83–3.46, p < 0.001). This effect remains also after adjusting for TNM stage (model 2: SHR 2.39, 95%CI 1.70–3.34, p < 0.001). Women with 1 or more geriatric characteristics had a cumulative incidence of other cause mortality of 90.7% (95%CI 83.9–94.7%) compared to those without geriatric characteristics: 74.6% (95%CI 65.7–81.5%) (Fig. 1c. Model 1: SHR 2.06, 95%CI 1.55–2.24), p < 0.001). After adjusting for TNM stage (Model 2: SHR 1.97, 95%CI 1.46–2.63, p < 0.001) and additionally for age (Model 3: SHR 1.66, 95%CI 1.23–2.25, p < 0.001) similar effects were seen. With 2 or more comorbidities the cumulative incidence of other cause mortality was 90.3% (95%CI 82.7–94.7%) versus 77.2% (95%CI 69.2–83.3%) in with 0–1 comorbidities (Fig. 1.d. Model 1: SHR 1.72, 95%CI 1.31–2.28, p < 0.001). Also after adjusting for TNM stage (Model 2: SHR 1.60, 95%CI 1.20–2.12, p = 0.001) and additionally for age (Model 3: SHR 1.47, 95%CI 1.11–1.96, p = 0.007) similar effects were seen.

When combining both age and geriatric characteristics, we observed higher cumulative incidences of other cause mortality in those aged over 80 years without geriatric characteristics (cumulative incidence 87.9%, 95%CI 77.4–93.8%. Model 2: SHR 2.32, 95%CI 1.56–3.45, p < 0.001), those aged 65–79 years with 1 or more geriatric characteristic (cumulative incidence 79.7, 95% CI 57.9–91.0%. Model 2: SHR 2.46, 95%CI 1.16–5.22, p = 0.019) and those aged over 80 years with 1 or more geriatric characteristics (cumulative incidence 92.8%, 95%CI 85.6–96.4%. Model 2: SHR 3.35, 95%CI 2.24–5.01, p < 0.001) compared to those aged 65–79 years without geriatric characteristics (cumulative incidence 57.5%, 95%CI 43.1–69.5. SHR 1.00 reference) (Fig. 1e).

When combining age and comorbidity, we observed higher cumulative incidences of other cause mortality in those aged 65–79 years with 2 or more comorbidities (cumulative incidence 77.3, 95% CI 53.7–89.8%. Model 2: SHR 1.76, 95%CI 0.95–3.26, p = 0.071), those aged over 80 years with 0–1 comorbidities (cumulative incidence 88.7%, 95%CI 79.7–93.9%. Model 2: SHR 2.49, 95%CI 1.63–3.80, p < 0.001), and those aged over 80 years with 2 or more comorbidities (cumulative incidence 93.0%, 95%CI 85.1–96.8%. Model 2: SHR 3.11, 95%CI 2.14–5.12, p < 0.001) compared to those aged 65–79 years with 0–1 comorbidities (cumulative incidence 59.5%, 95%CI 45.5–71.0. SHR 1.00 reference) (Fig. 1f).

Table 3 shows the reasons for omitting surgery in women treated with PET. For roughly half of the women (52.9%) no information was found in medical charts. In 17.1% it was stated in the medical chart that the patient had a preference for PET over primary surgery. In 11.7% surgery was omitted due to the patient’s physical condition and in 4.2% the patient’s mental condition was the reason for omission of surgery. In 5.1% of women inoperability was noted, but it was not specified whether this was due to the women’ condition or due to inoperability of the tumour. Age alone was given as a reason in 3.5% of women.

**Table 3:** Reasons to omit surgery in women with non-metastatic breast cancer treated with ET.

	N=	%
No information	136	52.9
Wish patient	44	17.1
Physical conditon	30	11.7
Mental condition	11	4.2
Mental and physical condition	2	0.8
Inoperable	13	5.1
Age only	9	3.5
Other diagnosis determines prognosis	4	1.6
Other (reasons noted: tumor was chance finding, due to surgery, no primary tumor, not opportune according to surgeon, pleural effusion/axillary nodes)	8	3.2

In case another reason e.g. “mental condition” was mentioned together with “wish patient” this was counted as the first reason in this table

## Discussion

In this study, we showed the real-life trajectories of women that were treated with PET between 1997 and 2004. We showed that the ten year cumulative incidence of distant metastasis in women treated with PET is very low and other cause mortality is high. As expected, our data confirm that in addition to increasing age, geriatric characteristics and comorbidity are predictors for other-cause mortality in older women with stage I-III breast cancer treated with PET.

These results underline the importance of estimating the risk of other cause mortality when counselling older women with non-metastatic breast cancer about treatment options. A full comprehensive geriatric assessment, has been shown to be strongly predictive of other-cause mortality in many previous studies [16–18]. Therefore, our results, based on a limited set of retrospectively obtained geriatric characteristics, might even underestimate the actual predictive capacity of these factors for other cause mortality.

It could be argued that PET is a good treatment option for frail older women with a short life expectancy. Previous randomized clinical trials have shown that PET is non-inferior to surgery with regard to overall survival for women over the age of 70, but a significant proportion of the study participants did require secondary surgery during follow-up. These trials were however performed with tamoxifen while in recent years, aromatase inhibitors have been shown superior to tamoxifen in both (neo)adjuvant and metastatic settings [19, 20] and more lines of endocrine therapy have become available. PET may therefore be more effective than what has been previously published. However, an observational study of women aged 80 years and older with stage I-II breast cancer showed that omission of surgery negatively impacted overall mortality after five years [21]. Furthermore, breast cancer surgery is generally regarded as minor surgery with a small risk of severe complications. However, in one of our previous studies of women aged 65 years or older, the risk of less severe post-operative complications, such as wound infections, hematomas and seromas was 19%, and was increased with older age and comorbidities. There were no severe life-threatening complications and mortality was not higher in the group of older women with complications [13]. Furthermore, endocrine therapy also comes with side-effects, with 31 to 73% of women discontinuing adjuvant endocrine therapy within 5 years after initiation, mostly because of toxicity [22]. Our group recently investigated toxicity of adjuvant endocrine therapy in older patients with breast cancer and its impact on quality of life. The results showed that patients who discontinued therapy due to toxicity had a worse quality of life compared to patients who continued therapy. However, discontinuation of treatment did not positively affect quality of life after discontinuation, which implies that the poorer quality of life is not caused by toxicity [23]. Although these studies have not been performed in women treated with PET, is it likely that side-effects occur as frequently in this group of women. Knowledge on these real-life treatment trajectories, side-effects and complications might aid older women with breast cancer and their physicians, so the impact of primary surgery versus PET (with possible side-effects and the need for chronic medication use) can be weighed for their preferred treatment.

Recently, a prediction tool was developed in the British Age Gap study to aid decisions for primary treatment in women with breast cancer aged 70 and older [10]. This model could help determine which older women might benefit most from PET or from primary surgery by determining other cause mortality and breast cancer-specific mortality. The researchers also investigated the impact of decision tools itself on quality of life in a randomized trial, but no difference in quality of life was seen between women that used the decision tool versus those receiving usual care. Interestingly, the fraction of treatment with PET was higher in those using the decision tool than those receiving usual care (21.0 versus 15.4 per cent) [24]. Furthermore, the British Age Gap study showed that quality of life dropped significantly between baseline and the first six weeks in both treatment groups, which did not recover within the first two years [25]. As for the functional status, a gradual decline was observed for women treated with PET, while women who received primary surgery showed an early sharp fall between baseline and 6 weeks. Both groups failed to recover to their baseline functional status. In contrast, a prospective cohort study from our group (in preparation) into older women with early-stage breast cancer with questionnaires including the Groningen Activity Rating Scale (GARS) and the EORTC-QLQC30 and BR23 showed that functional status and quality of life did not decline after breast surgery, irrespective of the occurrence of postoperative complications, which suggests that the impact of postoperative complications on these outcomes are in fact minimal. Hence, the decision for PET or surgical treatment remains a personal decision but the risks of surgery appear to be low, even in women with geriatric characteristics present. It is noteworthy that in more than half of our patients no information was recorded on why primary endocrine therapy was chosen over surgery, which is comparable to the results of Hamaker et al. [3]. Probably there are several reasons to choose PET within this group, which might be or not be related to frailty. Recording reasons for treatment decisions more often would aid future research and ultimately provide information to aid personal treatment decisions for older patients. Also, for a large proportion of our patients that were treated with PET no information on hormone receptor status was available, and around 5% were treated with PET despite negative hormone receptor expression. This partly reflects the time period in which hormone receptor expression was not routinely assessed, but another hypothesis would be that the omission of hormone receptor testing might be associated with frailty. However, with the data from this cohort these hypotheses cannot be adequately tested.

Strengths of this study include the real-world data with registration data, which includes all consecutive women aged 65 and over diagnosed with breast cancer in the region West in the Netherlands over 8 years. Therefore, there was no selection in women included. Another strength is the analysis of competing other cause mortality and the long-term follow-up data that were available. Limitations of this study include its retrospective design and the associated missing data on cause of death and reasons for treatment decisions. Furthermore, the number of geriatric characteristics measured is limited, and was dependent on what was noted by medical personal in the charts. In addition, the incidence of distant metastasis in women treated with PET may be underreported due to loss to follow up of frail women. Therefore, this study might overestimate the incidence of other cause mortality.

In conclusion, the ten year incidence of other cause mortality in older women treated with PET increases with age, the presence of geriatric characteristics and comorbidity, and is much higher than the risk of distant metastasis. This underlines the importance of predicting other cause mortality when counselling older women with non-metastatic breast cancer in their treatment options. In order to more accurately identify which older women benefit from a certain treatment option, more geriatric-based prognostic factors should be included in decision making in daily practice, for example by including screening tools or a comprehensive geriatric assessment.

## Electronic supplementary material

Below is the link to the electronic supplementary material.

Supplementary Material 1

## Abbreviations

PET: primary endocrine therapy
